# The efficacy and safety of Chinese herbal medicine Shen-Qi Hua-Yu formula in patients with diabetic lower extremity artery disease: Study protocol of a multi-center, randomized, double-blind, placebo-controlled trial

**DOI:** 10.1097/MD.0000000000018713

**Published:** 2020-01-17

**Authors:** Yulin Leng, Hong Gao, Xiaoxu Fu, Hongyan Xie, Zhipeng Hu, Jianwei Zhu, Xiaoke Liu, Xiujuan Zhou, Ziyan Xie, Chunguang Xie

**Affiliations:** Hospital of Chengdu University of Traditional Chinese Medicine, PR China.

**Keywords:** Chinese herbal medicine formulas, lower extremity artery disease, randomized controlled trial, Shen-Qi Hua-Yu formula, type 2 diabetes mellitus

## Abstract

**Background:**

Lower extremity artery disease (LEAD) is greatly harmful to Type 2 Diabetes Mellitus patients. Traditional Chinese Medicine (TCM) is an alternative therapy to delay the development of macrovascular diseases, but the existing evidence of its efficacy, safety and mechanism of action is insufficient. We report a study protocol of a multi-center, randomized, double-blind, placebo-controlled trial that aims to use well-designed clinical trial to evaluate the efficacy and safety of Chinese herbal medicine (CHM) Shen-Qi Hua-Yu formula, and to explore efficacy mechanism of the TCM granules and the biomarkers of TCM syndrome.

**Methods:**

This is a multi-center, double-blind, randomized, and placebo-controlled study that randomized 120 participants into 2 groups. The treatment group will receive TCM granules and conventional medicine, while the control group will receive placebo in addition to conventional medicine. Two groups will receive 12-week treatment and 48-week follow-up, with a total of 13 visits. Primary efficacy outcomes included ankle brachial index. Secondary efficacy outcomes included fasting plasma glucose, blood lipid, hemorheology indexes, advanced glycation end products, the inner diameter, peak systolic velocity, end diastolic velocity and mean average velocity of the anterior tibial artery, posterior tibial artery and dorsalis pedis artery, and TCM syndrome score. The safety and endpoint outcomes will be evaluated in this trial. The study will explain the biological therapeutic mechanism of Shen-Qi Hua-Yu formula for diabetic LEAD, and try to use Isobaric tags for Relative and Absolute Quantitation (iTRAQ) and Western blot to screen biomarkers of characteristic diagnosis and clinical efficiency evaluation of the TCM syndrome.

**Discussion:**

This study is a multi-center, randomized, double-blind, placebo-controlled trial to evaluate the efficacy and safety of CHM in patients with diabetic LEAD, and to interpret the therapeutic mechanism of Shen-Qi Hua-Yu formula in treatment of diabetic LEAD through proteomics technology, and to screen biomarkers with characteristics of TCM diagnosis and clinical efficacy evaluation. On the other hand, to our knowledge, this study may be the first trial of CHM formulas to observe cardiovascular outcomes through long-term follow-up for the treatment of diabetic LEAD, which is of great value.

**Trial registration:**

This study is registered on the Chinese Clinical Trial Registry: ChiCTR1900026372.

## 1 Introduction

Lower extremity artery disease (LEAD) is the major cause of foot ulcer, amputation and even death in Diabetes Mellitus patients.^[1]^ It is a risk factor of cardiovascular and cerebrovascular diseases as well. The China DIA-LEAD Study investigated 10,804 diabetic patients, and found that LEAD is the most common major vascular complication of diabetes, and the overall prevalence rate is about 21.2%.^[2]^ It imposes huge medical and economic burden to patients and the society. The basic pathological change of LEAD is atherosclerosis. Compared with patients without diabetes, LEAD in diabetic patients are characterized by more extensive affected blood vessels, more dispersed atherosclerotic lesions, more severe lumen stenosis, and more prone to plaque rupture. The degree of arterial injury in these patients is characterized by early onset, severe condition and poor prognosis.^[2,3]^

In recent years, a number of clinical studies have shown that the effect of intensive glucose control in macrovascular disease is continuing uncertainty.^[4–7]^ The cause of this phenomenon might be diabetic vascular injury attributed to various factors. Currently, the treatment of this disease needs to comprehensively evaluated and controls the risk factors that might cause LEAD injury, such as hyperglycemia, hypertension, dyslipidemia, etc. Although the clinical efficacy of statins and aspirin in the treatment of LEAD has been determined, the use of multiple drugs in combination increases the cost of medical care and tends to cause adverse reactions between drugs. The main treatment of diabetic LEAD is to control hyperglycemia, regulate dyslipidemia and stabilize atherosclerotic plaque with statins, and inhibit platelet with aspirin. The search for a comprehensive holistic approach to effectively delay or even prevent the development of new drugs for LEAD has never stopped.

In China, many patients with diabetic LEAD look for Chinese herbal medicine (CHM) formulas for treatment. In the theory of Traditional Chinese Medicine (TCM), the incidence of diabetes is fundamentally due to damage of body. The decline of body's function can produce hidden pathogenic factors such as blood stasis and continuously damage arteries. At present, this concept has been widely recognized by TCM doctors and researchers. These pathogenic factors do not have obvious manifestations in the early stage of disease. With the accumulation of pathogenic factors, the continuous abnormality of clinical indicators such as hyperglycemia and dyslipidemia eventually lead to artery injury. Blood stasis is the key pathological factor in development of diabetic LEAD. In the system of TCM, for “blood stasis” material basis research thinks, “blood stasis” contains the abnormal state of hemorheology and prethrombotic state and thrombosis, etc. It will damage the vessel wall, resulting in thickening of basilar membrane, narrowing of lumen and even blockage. CHM is divided into those that can strengthening healthy or dispel evil. Many of them have the effect of removing blood stasis. Tonifying CHM is helpful to restore the function of the body. Blood-activating and stasis-eliminating CHM is helpful to remove the stimulation of harmful factors. Therefore, it has a broad prospect to search for TCM in diabetic LEAD treatment.

Our research group has completed many national and provincial scientific research projects, a total of more than 10 studies. A large number of basic research and clinical trials prove that Shen-Qi series formulas are evidently efficient in the prevention and treatment of diabetic artery injury. We has conducted a randomized controlled trial (RCT) which is regards 120 patients with diabetic angiopathy as object (National Traditional Chinese Medicine Clinical Research Base building scientific research funding project, JDZX2012134).^[8]^ After 8 weeks of conventional hypoglycemic treatment combined with Shen-Qi series formulas in the treatment group, their TCM syndrome score, fasting plasma glucose (FPG), 2h plasma glucose (PG), and glycosylated serum protein were lower than the control group, and the life quality score was higher than that before treatment (*P* < .01), and the total effective rate was about 94.7%. We used Shen-Qi series formulas for RCT of diabetic patients (the project from the State Administration of Traditional Chinese Medicine, JDZX2015212), and after 9 months of follow-up, we found that as early as possible use of Shen-Qi series formulas in diabetic patients can be widely used to improve the whole blood viscosity, plasma viscosity, erythrocyte rigidity index, erythrocyte deformation index, erythrocyte aggregation index, fibrinogen, improve the prethrombotic state, and delay the occurrence and development of LEAD.

A meta-analysis of RCTs of Shen-Qi series formulas in the treatment of type 2 diabetic angiopathy showed that, compared with the conventional hypoglycemic agent group, Shen-Qi series formulas combined with hypoglycemic agent for the treatment of diabetic angiopathy can improve the efficiency rate, and reduce FPG, 2hPG, C-reactive protein and Leptin, as well as improve adiponectin, but it was also found that the quality of the included studies was limited.^[9]^ Therefore, it is necessary to carry out strictly designed large-scale samples, multi-center, randomized, controlled and double-blind clinical research, so as to provide high-quality evidence of evidence based medicine (EBM) for the efficacy and safety of TCM treatment of diabetic LEAD.

In the theory of TCM, the essence of diabetic LEAD is deficiency of Qi-yin and blood stasis in blood vessels. What is innovative and advantageous about this study is that it is based on syndromes differentiation of the disease. Compared with improving hyperglycemia, this approach focuses on restoring the body's own functional state.

The clinical evaluation system of the study is consists of the following five aspects: We will conduct dynamic evaluation of the efficacy of Shen-Qi Hua-Yu formula in diabetic LEAD from aspects of macrovascular protection efficacy index, endpoint event, TCM syndrome efficacy and patients’ comprehensive quality of life, and monitor the whole process of clinical intervention from the safety of TCM prescription. The study will comprehensively evaluate the intervention of Shen-Qi Hua-Yu formula in diabetic LEAD from the above 5 aspects, and establish a clinical efficacy evaluation method that reflect the characteristics of TCM prevention and treatment.

The mechanism of TCM diagnosis and treatment needs to be clarified from the aspects of symptoms, signs and material basis, especially the last one. Therefore, on the basis of EBM, the study will use Isobaric tags for Relative and Absolute Quantitation (iTRAQ), Western blot to explain the biological therapeutic mechanism of Shen-Qi Hua-Yu formula in the treatment of diabetic LEAD, and try to screen biomarkers of characteristic diagnosis and clinical efficiency evaluation of the TCM syndrome.

The purpose of this clinical study is:

(1) to evaluate whether Shen-Qi Hua-Yu formula is effective and safe for lower extremity artery lesions in diabetic LEAD patients;
(2) to elucidate the biological therapeutic mechanism of Shen-Qi Hua-Yu formula for diabetic LEAD through proteomics techniques;
(3) to screen biomarkers with the characteristics of TCM diagnosis and clinical efficiency evaluation.

## 2 Methods

### 2.1 Design

**Figure 1:**
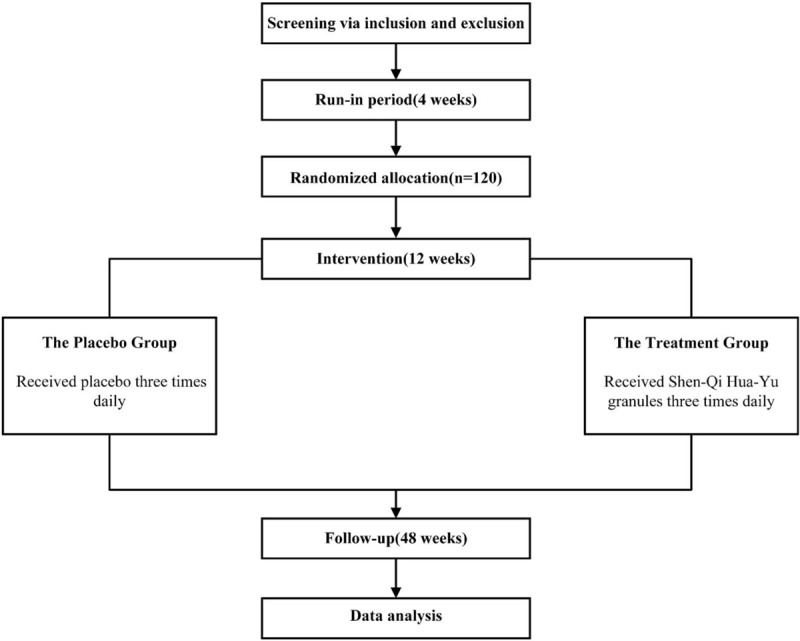
Flow chart of the clinical trial.

This study is a randomized, placebo-controlled, multi-center and double-blind clinical trial. The study protocol follows the recommendations of the Standard Protocol Items for Randomized Trials and the Consolidated Standards of Reporting Trials Extension for CHM Formulas statement.^[10,11]^ The study is registered on Chinese Clinical Trials.gov with approval number ChiCTR1900026372. The study will be carried out according to the following flow: First of all, screening via inclusion and exclusion criteria will lead into the run-in period; after a 4-week's run-in period, participants’ blood glucose, blood lipid and blood pressure must meet the standard before entering the treatment period; randomization; a treatment period of 12 weeks; follow-up observation will continue once every 12 weeks until 48 weeks after treatment. The research flow chart is shown in Figure 1.

### 2.2 Study visit

In this study, participants qualified for inclusion in this trial will be selected at the first visit, and will be well-prepared for the run-in period at the second visit, at last will be randomly assigned to TCM granules or placebo at the third visit. During the treatment period, participants will receive outpatient and telephone visits every 2 weeks. At the 2nd, 6^th^, and 10th weeks of treatment, the researchers will assess the participant's response to the drug through telephone visit. At the 4th, 8th, and 12th weeks of treatment, researcher will visit participants at the research settings and be evaluated according to the research schedule. After the end of the treatment period, participants will be followed up four times at the 12th, 24th, 36th, and 48th weeks. The researchers will evaluate the changes in the outcome of lower extremity artery effective measures from baseline. Each participant will be visited 13 times in this study.

### 2.3 Diagnostic criteria

#### 2.3.1 Western medicine (WM) diagnostic criteria

The diagnostic criteria for diabetes is the WHO diagnostic criteria in 1999,^[12]^ and diagnostic criteria for LEAD refer to the consensus for the management of peripheral surgical disease.^[13]^ The following 2 criteria must be met simultaneously:

(1) typical diabetes symptoms and random venous PG ≥ 11.1 mmol/L or FPG ≥ 7.0 mmol/L or in an oral glucose tolerance test, 2hPG ≥ 11.1 mmol/L, or have been diagnosed as diabetes in the past;
(2) conform to diagnosis of LEAD, that is, resting ankle brachial index (ABI) ≤0.90, or feel discomfort during exercise and resting ABI ≥ 0.90, such as ABI decreased by 15% −20% after bicycle ergometer test, or resting ABI < 0.40 or ankle blood pressure <50 mmHg or toe blood pressure <30 mmHg, or resting ABI > 1.3 and toe brachial index <0.65 or lower extremity vascular doppler shows abnormalities such as atherosclerotic plaque.

#### 2.3.2 TCM diagnostic criteria

Referring to “Guiding principles for new drug clinical research of TCM”, the signs and symptoms of Qi-yin deficiency with blood stasis syndrome are as follows: thirst, weakness and tiredness, disinclined to talk, palpitation, insomnia, numbness or pain of limbs, legs with deep purple skin, chest distress or stabbing pain, tortuous sublingual veins, pale tongue with purple speckles with white fur, wiry and unsmooth pulse.^[14]^

### 2.4 Inclusion criteria

(1) Diagnosis of LEAD of diabetes as well as Qi-yin deficiency with blood stasis syndrome according to WM and TCM.
(2) According to Fontaine stage^[15]^ of LEAD, patients with stage I, II a, and II b are eligible.
(3) Aged between 35 and 75 years old.
(4) Glycated hemoglobin ≤9.0%.
(5) Women of childbearing age are required negative pregnancy test and agree to use reliable contraception measures during the study.
(6) Voluntarily participate in the test and sign the informed consent.

### 2.5 Exclusion criteria

(1) Patients with cardiac and renal insufficiency, acute coronary syndrome, cerebrovascular accident, vasculitis, venous dysfunction of lower limbs, hematopoietic system disorders and mental diseases.
(2) Patients with acute metabolic disorders such as diabetic ketoacidosis within the past 1 month.
(3) Severe infection in the past 1 month.
(4) Patients with allergic constitution.
(5) Uncooperative in run-in period.
(6) Having a history of alcoholism or drug abuse.
(7) Having a history of another clinical study in the previous 3 months.
(8) Patients considered by the investigator to be unfit for this study.

### 2.6 Recruitment

There are 3 comprehensive hospitals involved in this study and participants will be recruited through posters at each participating center. The recruitment and research settings containing Hospital of Chengdu University of Traditional Chinese Medicine, Meishan Hospital of TCM, and Shuangliu area hospital of TCM. It is worth mentioning that, the primary sponsor is the Hospital of Chengdu University of TCM. It is the National TCM Clinical Research Base focusing on diabetes, the national international cooperation and exchange base of TCM, the National Clinical Trial of Chinese Medicine.

### 2.7 Randomization

The randomization sequence will be generated with the PROC PLAN procedure statements of SAS software package by the statistician experts of Sichuan University, and will be concealed and disseminated using opaque envelopes.

### 2.8 Blinding

In this study, researchers, participants and outcome assessors will be blinded during the trial period. Unblinding can only be performed when patient's adverse events needs to be evaluated. When an emergency occurs, the researcher should immediately inform the primary researcher. The unblinded participant will be eliminated from the study. In this study, intentional analysis will be applied to the unblinding participants. The researcher should record the reason, time and place in Case Report Forms. Statistician experts and drug management staff are not directly involved in the trial process.

### 2.9 Interventions

#### 2.9.1 Run-in period

All participants will be treated with metformin and/or alpha-glucosidase inhibitor and/or insulin to control hyperglycemia, angiotensin-converting enzyme inhibitors (ACEI) and/or angiotensin receptor blockers (ARB) and/or calcium channel blockers (CCB) and/or Thiazide-like diuretic to control hypertension, aspirin to inhibit platelet and atorvastatin to regulate dyslipidemia before entering the treatment period. After a 4-week's run-in period, participants’ blood glucose, blood lipid and blood pressure must meet the following criteria before entering the treatment period. If people under the age of 65 years, the following criteria must be met:

(1) FPG between 4.4 mmol/L and 7.0 mmol/L, and non-fasting blood glucose <10.0 mmol/L;
(2) blood pressure ≤130/80 mmHg;
(3) low-density lipoprotein cholesterol ≤2.6 mmol/L.

If people over the age of 65 years, the following criteria must be met:

(1) FPG between 5.0 mmol/L and 8.3 mmol/L, and non-fasting blood glucose <11.1 mmol/L;
(2) blood pressure ≤140/90 mmHg;
(3) low-density lipoprotein cholesterol ≤2.6 mmol/L.

#### 2.9.2 TCM intervention

The study has 2 arms. The WM conventional treatment of controlling hyperglycemia contain metformin and/or alpha-glucosidase inhibitor and/or insulin. ACEI and/or ARB and/or CCB and/or Thiazide-like diuretic are selected to control hypertension, aspirin to inhibit platelet and atorvastatin to control hyperlipidemia. After enrollment, participants will be randomized to either groups:

(1) the treatment group (n = 60), participants will take CHM Shen-Qi Hua-Yu granules, which is composed of Renshen (Ginseng), Zhihuangqi (Astragalus membranaceu), Shanzhuyu (Asiatic Cornelian Cherry Fruit), Shengdihuang (Unprocessed Rehmannia Root), Shuizhi (Leech), Dilong (Earthworm), Jixueteng (Suberect Spatholobus Stem), Taoren (Peech Seed), Honghua (Carthamus Tinctorius), Niuxi (Twotoothed Achyranthes Root);
(2) the placebo group (n = 60). The action of each herb is summarized in Table 1. The treatment course is 12 weeks.

**Table 1:**
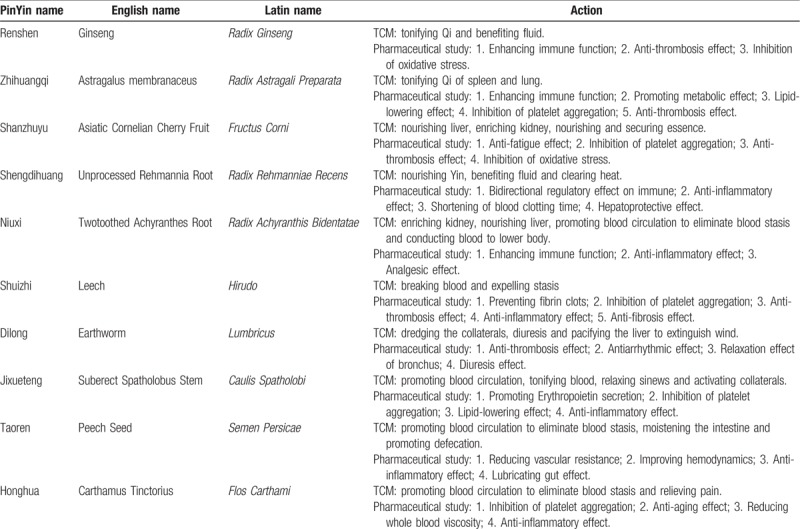
The action of each Traditional Chinese Medicinal herb.

In order to stabilize the formulation, increase the convenience of taking medicine, remove the influence factors such as the method of decocting and the origin of medicinal materials, and facilitate the observation and blind implementation, the study adopted the preparation of granules. The placebo is made from dextrin, food colorants, bitters, and water. Placebo and Shen-Qi Hua-Yu granules are close to the same in color, smell, taste, appearance, packaging, and tag. They are prepared by Sichuan green pharmaceutical technology development Co. Ltd, Sichuan, China.

Participants will dissolve granules into 100 ml of hot water, cover and seal for 3 to 5 minutes, then take it between 30°C and 36°C. Each time 1 bag of granules, 3 times a day.

### 2.10 Outcome measures

#### 2.10.1 Primary efficacy outcomes

ABI will be measured before treatment and after 12-week treatment and every 12 weeks during follow-up observation, 6 times in total. ABI will be measured and calculated using blood pressure cuff and continuous waveform doppler probe.

#### 2.10.2 Secondary efficacy outcomes

The color Doppler ultrasound will detect the inner diameter, peak systolic velocity (PSV), end diastolic velocity (EDV) and mean average velocity (Vm) of the anterior tibial artery, posterior tibial artery and dorsalis pedis artery of the patients will be detected. The pulse index (PI) and resistance indices (RI) will be calculated according to the formula. PI = (PSV-EDV)/Vm, RI = (PSV-EDV)/PSV. It will be measured before and after 12 weeks of treatment and every 24 weeks during the follow-up observation, 4 times of measurement in total.

Other secondary efficacy outcomes include changes in FPG, total cholesterol, triglycerides, high-density lipoprotein cholesterol, low-density lipoprotein cholesterol, homocysteine, hemorheology indexes (plasma viscosity, whole blood viscosity, hematocrit, erythrocyte aggregation index, erythrocyte rigidity index, fibrinogen, erythrocyte sedimentation rate, equation K value of erythrocyte sedimentation rate), advanced glycation end products, and TCM syndrome score measured by questionnaire. All of these will be measured before and after 12 weeks of treatment, 2 times in total. Three to 4 ml venous blood will be collected for each detection.

#### 2.10.3 Therapeutic mechanism outcomes

The study will use iTRAQ to quantify detection and analyze the serum protein of participants before and after treatment and then determine it by western blot.

#### 2.10.4 Endpoint outcomes

The endpoint incidents are defined as major adverse cardiovascular events that including cardiovascular death, myocardial infarction and ischemic stroke, or complete occlusion of the blood vessels, or ulcer, or gangrene of the lower limbs. In this study, the incidence of endpoint incident and the time of progression to endpoint incident will be selected as the indicators.

#### 2.10.5 Safety outcomes

Safety will be monitored using blood, urine and stool routine examination, liver function (alanine aminotransferase, aspartate aminotransferase, total bilirubin, alkline phosphatase, glutamyl transpeptidase), renal function (blood urea nitrogen, creatinine) and electrocardiogram. The researchers should record the occurrence and nature of each participant's pre-existing conditions, including clinically significant signs and symptoms of the disease being treated during the study. All adverse events related to medicine will be immediately reported in written Case Report Forms to the Ethical Review Committee of Hospital of Chengdu University of Traditional Chinese Medicine and each participating center.

### 2.11 Sample size

The study is an optimality trial, and the control group is placebo group. ABI is the primary outcome, and setting the optimum value to 0.04 based on the response rates of treatment. According to previous clinical research data, the mean value between the 2 groups is 0.13, and the standard deviations of treatment group and control group are 0.19 and 0.11 respectively. Set α to 0.025 and β to 0.2. Used PASS 11 software to calculate the sample size of each group is 48. Meanwhile, taking into account a dropout of 20% and according to the formula that is n^ = n / (1–f), we concluded that in order to ensure statistically significant results, the total number of study samples is determined to be 120 cases with 60 for each group.

### 2.12 Statistical analysis

The statistical analysis of this study will be conducted by statisticians of the Sichuan University. They will use Statistical Analysis software packages SPSS 23.0 and SAS 9.1. Demographic and other baseline characteristics will analyze using the full analysis set (FAS). Full analysis set will analyze with intention-to-treat (ITT), and per-protocol set (PPS) will statistically analyzed. Safety analysis will be performed according to safety data set for subjects who receive at least 1 treatment after randomization. Normally distributed data is represented by mean ± standard deviation, and non-normally distributed data is represented by median and interquartile range. Comparisons between groups of measurement data will be conducted by using independent-sample *t* test, and within group differences will be tested with paired *t* test. Differences between groups of numeration data will be assessed with chi-square test. Comparison of survival rate and survival time between groups will be analyzed by Kaplan–Meier curve to explore each possible relevant factor. Covariates were then included in the multi-factor Cox regression model for analysis to detect the influence of these variables on end event of the study. This study is a multi-center clinical trial. CMH χ2 will be used for numeration data, analysis of variance (ANOVA) and an analysis of covariance (ANCOVA) will be used for measurement data, and log-rank test will be used for endpoint outcomes. After deducting the influence of polycentric effect, comparing efficacy outcomes, endpoint outcome index and safety outcomes between 2 groups.

### 2.13 Data management

Case Report Forms identified by sub-centers are used for data acquisition. The primary researcher saves the individual participant data (IPD). Electronic Data Capture system of ResMan is used for data management. IPD will be shared within 6 months after this study complete. The way of sharing data is public accessible via ResMan software.

### 2.14 Ethics and dissemination

The study protocol was approved by the Ethical Review Committee of Hospital of Chengdu University of Traditional Chinese Medicine (Chengdu, China). Each participant will voluntarily participate in the trial and sign informed consent.

## 3 Discussion

At present, there are many clinical trials conducted to evaluate the efficacy of TCM for diabetic LEAD. TCM has multiple advantages over the prevention and treatment of diabetic LEAD, such as overall regulation, co-regulation of blood glucose and lipids, and improvement of patients’ quality of life. Pu et al. conducted a RCT on the efficacy and safety of TCM Jiangtangtongmai capsule in the treatment of diabetic macroangiopathy. The results showed that TCM Jiangtangtongmai capsule can thinning carotid intima-media thickness and reduce the area of arterial plaque.^[16]^ A study of meta-analysis evaluated the efficacy of Chinese herbal compounds in the treatment of diabetic macroangiopathy, which involving 20 RCTs of 1479 patients.^[17]^ The curative effect of Chinese herbal compounds combined with WM conventional treatment is better than that of WM conventional treatment. This treatment decreases the participants’ FPG, 2hPG, total cholesterol, low-density lipoprotein cholesterol, glycated hemoglobin and improves TCM symptom score. Liu et al conducted a RCT with Huoxuetongluo powder in the treatment of diabetic LEAD.^[18]^ The trial found that the powder have therapeutic effect in improving ABI and patients’ pain, coldness and numbness rating scale scores, as well as improving the stenosis of lower extremity artery. Nevertheless, there is no description of the method of allocation concealment and double-blind.

However, it is worth mentioning that most of the published clinical trials of TCM on diabetic LEAD are poor in methodology, with few strictly randomized and double-blind, and few of them have been followed up. The results are difficult to provide reliable and valuable evidence. TCM has accumulated rich experience in diagnosis and treatment in long-term clinical practice, but this is a subjective evaluation, which is considered as low-quality EBM evidence. Therefore, it is essential to conduct well-designed clinical study to provide high-quality EBM evidence. The trial is valuable for it may provide scientific and rigorous EBM evidence for the efficacy and safety of Shen-Qi Hua-Yu formula in treating diabetic LEAD, as well as exploring the therapeutic mechanism of TCM syndrome with the biomarkers through proteomics technology.

## 4 Trial status

Patient recruitment for the trial began in September 2019. Data collection will continue until the end of September 2021.

## Acknowledgments

We thank Weihong Li and Bohua Yan for their suggestions in the design of this trial, and statisticians of the Sichuan University for their support of statistical analysis. We appreciate the efforts and cooperation for research staffs of Meishan Hospital of TCM and Shuangliu area hospital of TCM in this trial.

## Author contributions

**Conceptualization:** Yulin Leng, Hong Gao, Chunguang Xie.

**Data curation:** Jianwei Zhu, Xiaoke Liu, Xiujuan Zhou, Ziyan Xie.

**Funding acquisition:** Chunguang Xie.

**Investigation:** Xiaoxu Fu, Hongyan Xie, Zhipeng Hu.

**Methodology:** Yulin Leng, Hong Gao, Chunguang Xie.

**Project administration:** Chunguang Xie.

**Resources:** Yulin Leng, Hong Gao, Chunguang Xie.

**Supervision:** Chunguang Xie.

**Writing – original draft:** Yulin Leng, Hong Gao.

**Writing – review and editing:** Chunguang Xie.
